# Hypothalamic-Pituitary-Adrenal Hormones Impair Pig Fertilization and Preimplantation Embryo Development via Inducing Oviductal Epithelial Apoptosis: An In Vitro Study

**DOI:** 10.3390/cells11233891

**Published:** 2022-12-02

**Authors:** Jin-Song An, Guo-Liang Wang, Dong-Ming Wang, Yong-Qing Yang, Jia-Shun Wu, Ying-Qi Zhao, Shuai Gong, Jing-He Tan

**Affiliations:** Shandong Provincial Key Laboratory of Animal Biotechnology and Disease Control and Prevention, College of Animal Science and Veterinary Medicine, Shandong Agricultural University, Taian 271018, China

**Keywords:** apoptosis, embryo development, HPA hormones, oviduct, pig

## Abstract

Previous studies show that stressful events after ovulation in sows significantly impaired the embryo cleavage with a significant elevation of blood cortisol. However, the effects of corticotropin-releasing hormone (CRH), adrenocorticotropic hormone (ACTH) and cortisol on fertilization and embryo development remain to be specified, and whether they damage pig embryos directly or indirectly is unclear. This study demonstrated that embryo development was unaffected when pig parthenotes were cultured with different concentrations of CRH/ACTH/cortisol. However, embryo development was significantly impaired when the embryos were cocultured with pig oviductal epithelial cells (OECs) in the presence of CRH/cortisol or cultured in medium that was conditioned with CRH/cortisol-pretreated OECs (CRH/cortisol-CM). Fertilization in CRH/cortisol-CM significantly increased the rates of polyspermy. CRH and cortisol induced apoptosis of OECs through FAS and TNFα signaling. The apoptotic OECs produced less growth factors but more FASL and TNFα, which induced apoptosis in embryos. Pig embryos were not sensitive to CRH because they expressed no CRH receptor but the CRH-binding protein, and they were tolerant to cortisol because they expressed more 11-beta hydroxysteroid dehydrogenase 2 (HSD11B2) than HSD11B1. When used at a stress-induced physiological concentration, while culture with either CRH or cortisol alone showed no effect, culture with both significantly increased apoptosis in OECs. In conclusion, CRH and cortisol impair pig fertilization and preimplantation embryo development indirectly by inducing OEC apoptosis via the activation of the FAS and TNFα systems. ACTH did not show any detrimental effect on pig embryos, nor OECs.

## 1. Introduction

Although it is known that stress can influence female reproduction, how stress affects embryo development is not very clear. Because there are reports that the early stages of pregnancy are more sensitive to stress than the later stages are [1,2], the preimplantation period is envisioned as one of the most stress-vulnerable phases [3,4]. However, reports on the effects of female stress on preimplantation embryos are very limited. Furthermore, although limited studies have demonstrated that restraint stress on pregnant mice significantly impaired preimplantation embryo development [5,6], how the stress effect is delivered to the preimplantation embryo is rarely reported [7,8]. In addition, whether the female stress would affect fertilization within the oviduct is not known.

Stresses are commonly associated with the enhanced activity of the hypothalamo-pituitary-adrenal (HPA) axis. The activation of the HPA axis leads to secretion of corticotrophin-releasing hormone (CRH), adrenocorticotropic hormone (ACTH) and glucocorticoids. For instance, restraint of female mice increased their CRH concentration in both serum and ovaries [9]. An immediate and constant increase in CRH secretion was observed following the transportation of ewes [10]. A significant elevation in serum corticosterone and cortisol was observed after mice were exposed to various stresses [11,12]. Moreover, sows exposed to stresses showed significant increases in cortisol concentrations [13]. However, the mechanisms by which the HPA hormones influence embryo development remain to be explored.

It is known that reproduction in female pigs is sensitive to various stressors, which can activate the HPA axis and facilitate the secretion of CRH, ACTH and cortisol [14,15]. Furthermore, repeated ACTH stimulation [16] or food deprivation [17] after ovulation in sows significantly impaired cleavage of the preimplantation embryos while inducing a significant elevation in the level of blood cortisol. However, although these data suggest that stresses might compromise the preimplantation embryo development by facilitating production of the HPA hormones in pigs, the mechanisms by which HPA hormones impair embryo development are largely unclear. Because of the high incidence of polyspermy during pig in vitro fertilization [18], the effects of oviducts on polyspermic fertilization should be studied in this species to find out the etiology for the pathological condition of polyspermy. Furthermore, although by using an in vitro model, Tan et al. [8] have observed that CRH and corticosterone affected mouse preimplantation embryo development indirectly by triggering apoptosis of the oviductal epithelial cells (OECs), their conclusions remain to be verified in other species.

Thus, the major objectives of this study were to specify the effects of CRH, ACTH and cortisol on pig embryo development; to clarify whether these hormones would damage pig embryos directly or indirectly by changing the oviductal environment; and to explore whether they would affect fertilization in the oviducts.

## 2. Methods

The procedures for animal care and handling were performed according to guidelines approved by the Animal Care and Use Committee of the Shandong Agricultural University P. R. China (Permit number: SDAUA-2001-001). Unless pointed out otherwise, all chemicals and reagents used in this study were bought from Sigma-Aldrich Corp. (St. Louis, MO, USA).

### 2.1. Collection of Ovaries and Oviducts

Pig ovaries and oviducts were obtained from the Feicheng slaughterhouse of Yinbao Food Corporation Ltd. (Taian, China). Only ovaries and oviducts at the follicular stage were collected from gilts, approximately 6 months after birth (approximately 100 kg bodyweight). While the ovaries were transported in a thermos bottle with sterile saline containing 100 IU/mL penicillin and 0.05 mg/mL streptomycin, maintained at 30–35 °C, the oviducts were transported in physiological saline solution, maintained at 4 °C, to the laboratory within 3 h after slaughtering.

### 2.2. Collection, In Vitro Maturation and Activation of Oocytes

To collect cumulus-oocyte complexes (COCs), 3–6 mm follicles were aspirated using a syringe containing Dulbecco’s phosphate-buffered saline (D-PBS, HyClone, Logan, UT, USA) supplemented with 0.88 mM CaCl_2_·2H_2_O, 0.49 mM MgCl_2_·6H_2_O, 0.1% polyvinyl alcohol, 0.03 mM phenol red, 50 IU/mL penicillin and 50 µg/mL streptomycin. The COCs recovered were washed three times in D-PBS, and those showing a uniform ooplasm and compact cumulus were selected for in vitro maturation. Approximately 80 ovaries were obtained on each experimental day, and the oocytes recovered from them were pooled and divided into different treatments.

The culture medium used for oocyte maturation was TCM-199 (Gibco, Grand Island, NY, USA) containing 10% porcine follicle fluid, 0.1% PVP, 0.91 mM sodium pyruvate, 3.05 mM glucose, 0.05 IU/mL FSH, 10 ng/mL EGF, 0.05 IU/mL LH, 0.57 mM cysteine, 50 μg/mL streptomycin and 100 IU/mL penicillin. We aspirated porcine follicular fluid from 3–6 mm follicles of the follicular stage ovaries. After centrifugation at 1600× *g* for 30 min, we collected the supernatants and filtered them sequentially through 0.22 μm syringe filters. The prepared follicular fluid was stored at −20 °C until use. We placed the maturation medium in culture wells of a 96-well plate (150 μL per well), and performed a pre-equilibration at 38.5 °C in an atmosphere of 5% CO_2_ in humidified air for 3 h before the introduction of the oocytes. We then washed the COCs three times in D-PBS and once in the maturation medium, and then, placed them in the wells (approximately 25 per well) covered with mineral oil before culture for 44 h at 38.5 °C under 5% CO_2_ in humidified air.

After the maturation culture, the cumulus cells were mechanically removed from oocytes by repeatedly pipetting in D-PBS with 0.1% hyaluronidase using a small-bore pipette. For activation treatment, the cumulus-free oocytes were first cultured for 5 min in D-PBS containing 5 µM ionomycin, and then, they were incubated for 5 h in PZM-3 medium with 2 mM 6-DMAP after being washed three times in PZM-3 medium.

### 2.3. Preparation of Oviduct Epithelium Cells (OECs) and Conditioned Medium (CM)

One oviduct was used on each experimental day. We dissected the oviducts free from surrounding tissues, ligated them at both ends, and washed them three times in Hanks’ balanced salt solution at 4 °C. After being closed off at one end with a clip, the oviduct was filled with 0.25% trypsin solution and closed off at the other end. Then, the oviduct was incubated at 37 °C for 45 min before being squeezed with tweezers to obtain the contents. We resuspended the recovered pellets in Dulbecco’s Modified Eagles Medium/Ham’s F12 (DMEM/F12, Gibco, Beijing, China) containing 10% (*v*/*v*) fetal calf serum (Gibco) and 0.5% (*v*/*v*) penicillin/streptomycin solution (Gibco) and washed them twice by centrifugation (200× *g*, 5 min). We added the final suspension (1~2 × 10^5^ cells/mL) to wells (500 μL per well) of a 24-well culture plate and cultured them at 38.5 °C in a humidified atmosphere of 5% CO_2_ in air. At 24 h after seeding, when most OECs had attached, the medium was renewed for the first time, and then, the medium was renewed every 48 h. Mouse OECs were prepared as reported previously [8].

Immunohistochemical detection of the cytokeratin filaments was conducted to check the purity of pig OECs we cultured. Pig OECs were cultured on glass coverslips in a 24-well dish. When cells grew to 20% or 90% of confluence, coverslips were rinsed with PBS and fixed in pure ethanol. Cytokeratin-18 were labeled using the cytokeratin-18 rabbit polyclonal antibody (1:100; 18708-1-AP, Proteintech, Chicago, IL, USA) and Cy3-conjugated goat anti-rabbit IgG (1:200; AP132C, Jackson ImmunoResearch, West Grove, PA, USA), and nuclei were stained with Hoechst 33324. Then, the sample was mounted, sealed and visualized under a fluorescent microscope. Each treatment contained three coverslips and three fields were observed on each coverslip. Approximately 200 cells were counted in each field to calculate rates of positive cells. The results showed that the cytokeratin 18-positive rates were 96.4 ± 2.0% and 98.2 ± 1.2% in OECs cultured to 20% and 90% of confluence, respectively.

To prepare CM, when the cultured OECs reached 60–70% of confluence, we replaced the spent DMEM/F12 with fresh serum-free DMEM/F12 without (control) or with CRH, ACTH or cortisol, and we cultured the growing OECs further for 48 h. Then, we replaced the medium with fresh PZM-3 medium without hormones and cultured cells for 24 h. After the culture, we collected the supernatants and centrifuged them for 10 min at 1000× *g* to collect CM. We froze and stored the CM in aliquots at −20 °C before use.

According to the experimental design, CRH, ACTH and cortisol and their antagonists were added to PZM-3 medium or to serum-free DMEM/F12 to observe their effects on the embryos and OECs, respectively. To prepare stock solutions, we dissolved CRH (10 mM) and ACTH (10 mM) in water, cortisol (10 mM) and RU486 (10 mM) in ethanol and antalarmin (10 mM) in dimethylsulfoxide (DMSO). We stored the stock solutions at −20 °C before use. When the OECs or embryos were treated with cortisol, RU486 or antalarmin, the control OECs/embryos were cultured with the same amount of ethanol or DMSO. The final concentrations of ethanol and DMSO in culture medium were 0.02–0.1% (*v*/*v*) and 0.02% (*v*/*v*), respectively.

### 2.4. Embryo Culture

Culture in PZM-3 alone: After the activation treatment, the oocytes were washed in D-PBS to remove 6-DMAP and were cultured in PZM-3 with different concentrations of CRH, ACTH or cortisol. Approximately 25 oocytes were cultured in 200 μL medium covered with paraffin oil in a well of a 96-well plate at 38.5 °C in 5% CO_2_ in air [19]. The medium was renewed every 72 h of culture. Culture wells containing fresh medium were precultured for 6 h under the same temperature and atmosphere conditions for equilibration. Then, embryos were transferred from the old wells to the equilibrated wells.

Coculture with OECs: When OECs grew to 70–80% confluent, the spent DMEM/F12 in wells of the 96-well plate was replaced with 200 μL of PZM-3 with or without CRH, ACTH or cortisol, and the OECs were cultured for 24 h. After the culture, approximately 25 activated oocytes were introduced into each well containing the precultured OECs, covered with mineral oil and cultured for 48 h. Then, the embryos were changed into the new precultured OECs every 48 h.

Culture in CM: Approximately 25 activated oocytes were cultured in 200 μL CM covered with mineral oil in wells of a 96-well plate. Then, the CM was renewed at 72 h intervals.

The cleavage rate was observed at 48 h of embryo culture, and the blastocyst rate was observed at 144 h of embryo culture under a phase contrast microscope. For cell number counting, some of the blastocysts were stained with Hoechst 33342 (10 μg/mL) and observed under a fluorescence microscope. For embryo culture, each treatment was repeated four times on four different experimental days with each replicate containing approximately 25 oocytes.

### 2.5. In Vitro Fertilization

Modified Tris-buffered medium (mTBM) was used for the fertilization medium, which contained 113.1 mM NaCl, 3.0 mM KCl, 20.0 mM Tris, 11.0 mM glucose, 5.0 mM Na-pyruvate, 7.5 mM CaCl_2_, 2 mM caffeine and 0.6% BSA. To prepare mTBM-CM, when the cultured OECs grew to 60–70% confluence, they were changed into serum-free DMEM/F12 without (control) or with CRH or cortisol and cultured further for 48 h. Then, the cells were cultured for 24 h in mTBM without hormones. At the end of the culture, we collected supernatants and centrifuged them for 10 min at 1000× *g* to obtain mTBM-CM. We froze and stored the mTBM-CM in aliquots at −20 °C before use. The freshly prepared mTBM or mTBM-CM was kept in 5% CO_2_ incubator for 10 h to stabilize its pH at 7.2 to 7.4.

After the maturation culture, we removed cumulus cells as described above, and oocytes showing the first polar bodies were washed three times in mTBM or mTBM-CM. After washing, 15 oocytes were placed in 50 μL mTBM or mTBM-CM under mineral oil in a well of a 96-well plate. The plate was kept in an incubator until spermatozoa were added for fertilization. Freshly-collected semen (1 mL) was washed once in D-PBS and once in mTBM or mTBM-CM by centrifugation at 200× *g* for 4 min. After the washing procedure, we resuspended the sperm pellets in 1 mL of mTBM or mTBM-CM and incubated them in a CO_2_ incubator at 38.5 °C for 1 h for capacitation. After capacitation and appropriate dilution with mTBM or mTBM-CM, we added 30 μL of the sperm suspension to 50 μL of the medium containing oocytes, producing a final concentration of 2.5 × 10^5^ sperm/mL. Then, the oocytes and spermatozoa were co-incubated for 6 h at 38.5 °C in an atmosphere of 5% CO_2_ in air.

After the co-incubation, oocytes were washed twice with pre-equilibrated PZM-3 medium and cultured for 10 h in PZM-3 (15 oocytes/150 μL medium) at 38.5 °C in an atmosphere of 5% CO_2_ in air. Then, the zona pellucida was removed by treating oocytes with 0.5% pronase for 2 min. The zona-free oocytes were fixed in 4% paraformaldehyde for 2 min and stained with 10 μg/mL Hoechst 33342 for 5 min before being mounted on a slide and examined for fertilization under a fluorescence microscope at a magnification of ×400. Oocytes with one or no pronucleus, oocytes with two pronuclei and those with three or more pronuclei were considered unfertilized, normally fertilized and polyspermic, respectively. Each treatment was repeated three times on three experimental days with each replicate including approximately 15 oocytes.

### 2.6. Hoechst Staining of OECs

Cultured OECs were stained with Hoechst 33342 in situ in wells of a 24-well culture plate. Briefly, at the end of culture, OECs were washed three times with PBS, and then, stained for 5 min in situ using 10 μg/mL Hoechst in the dark. Then, the stained cells were observed under a Leica DMLB fluorescence microscope at a magnification of 200×. The heterochromatin was heavily stained and was identified by its characteristic bright fluorescence. While the apoptotic cells showed pyknotic nuclei full of heterochromatin, healthy cells showed normal nuclei with sparse heterochromatin spots. Three to five fields were randomly observed in each well, and percentages of apoptotic cells were calculated from 200–250 cells observed in each field. For each experimental series, all images were acquired with identical settings. Cell numbers were counted on raw images by using the Image J software (1.51 K, National Institutes of Health, Bethesda, MD, USA). Briefly, the image was first converted into an 8-bit black and white format (image-Type-8-bit). The brightness threshold was then adjusted to eliminate the background brightness. Then, the threshold value for all the cells and for positive cells were set to 90–255 and 145–255, respectively. Finally, the range of nuclear area detected was set to 100 pixels. Each treatment was repeated three times on three different experimental days with each replicate including cells from one well of a 12-well culture plate.

### 2.7. Flow Cytometry Assay

The OECs were first stained with Annexin V-FITC/propidium iodide (PI) staining (556547; BD, Franklin Lakes, NJ, USA) and then measured by flow cytometry. Briefly, the OECs were washed in PBS and digested at 37 °C with 0.25% trypsin for 3–5 min. After that, a DMEM/F12 medium containing 10% fetal bovine serum was added to neutralize the residual trypsin. We resuspended the cells of each sample in 100 μL binding buffer, containing 5 μL Annexin V-FITC, and incubated them for 10 min at room temperature in the dark. Finally, we added 5 μL PI to the OECs and analyzed the fluorescence of 10,000 cells per sample using a LSRFortessa (BD). Each treatment was repeated three times on three different experimental days with each replicate including cells from one well of a 24-well culture plate.

### 2.8. Western Blot Analysis

We first prepared a radio immuno-precipitation assay (RIPA) buffer containing 150 mM NaCl, 1 mM phenylmethyl sulfonyl fluoride, 50 mM Tris (pH 8), 1.0% Triton X-100, 0.5% sodium deoxycholate, and 0.1% SDS. We then washed the OECs in cooled PBS, and lysed them in 100 μL RIPA buffer. After that, the total protein concentration was determined using a BCA Protein Assay Kit (P0012; Beyotime Institute of Biotechnology, Beijing, China) and was adjusted to 1 μg/μL. Then, 20 μL of total protein was placed in a 0.5-mL microfuge tube and frozen at −80 °C before use. To extract proteins, we added 5 μL of 5× sodiumdodecyl sulfate-polyacrylamide gel electrophoresis (SDS-PAGE) loading buffer to each tube and heated the tubes to 100 °C for 5 min. We ran SDS-PAGE on a 10% polyacrylamide gel. We transferred the proteins electrophoretically onto polyvinylidene fluoride membranes. After that, we washed the membranes in TBST (150 mM NaCl, 2 mM KCl, 25 mM Tris and 0.05% Tween 20; pH 7.4), blocked them with TBST containing 3% BSA at 37 °C for 2 h and incubated them at 4 °C overnight with primary antibodies. We then washed the membranes in TBST, and incubated them for 1.5 h at 37 °C with secondary antibodies. Finally, we detected signals using a BeyoECL Plus kit (P0018S, Beyotime). Each treatment was repeated three times on three different experimental days with each replicate including OECs from one well of a 6-well plate.

The primary antibodies we used are as follows: rabbit anti-activated caspase-3 antibodies (1:1000, ab2302, Abcam Co., Ltd., Beijing, China), goat anti-CRHR1 polyclonal antibodies (1:500, ab59023, Abcam Co., Ltd.), rabbit anti-GR antibodies (1:1000, ab196944, Abcam Co., Ltd.), rabbit anti-Fas (1:500, ab82419, Abcam Co., Ltd.), rabbit anti-TNFR (1:1000, AF8196, Beyotime Co., Ltd., Beijing, China), mouse anti-ACTB monoclonal antibodies (1:1000, CW0096, CWBio Co., Ltd., Beijing, China), and mouse anti-GAPDH monoclonal antibody (1:1000, CW0100, CWBio Co., Ltd.). The secondary antibodies are: HRP-conjugated rabbit anti-goat IgG (1:1000, A0181, Beyotime Co., Ltd.), goat anti-rabbit IgG (1:1000, CW0103, CWBio Co., Ltd.) and goat anti-mouse IgG (1:4000, CW0102, CWBio Co., Ltd.).

### 2.9. Real-Time PCR and Temperature Gradient PCR for OECs

RNA was isolated by treating the OECs with 1 mL TRIzol reagent, resuspended in diethyl pyrocarbonate-treated MilliQ water (DEPC-dH_2_O) and digested with RNase-free DNase I (Takara Biotechniques, Dalian, China). Spectroscopical quantification of RNA was performed at 260 nm. The RNA purity and integrity were assessed by determining the A260:A280 ratio (1.8–2.0) and performing electrophoresis in 1% agarose.

We conducted reverse transcription using a total volume of 20 μL and PrimeScript RT Reagent Kit (Takara RR047A): (a) we mixed 2 μL RNA sample, 2 μL 5× gDNA Eraser Buffer, 1 μL gDNA Eraser and 5 μL DEPC-dH_2_O in a 0.2-mL reaction tube; (b) we incubated the mixture at 42 °C for 2 min in a PCR instrument (Thermo Scientific, Hudson, NH, USA); (c) we added 4 μL 5× PrimeScript Buffer 2, 1 μL PrimeScript RT Enzyme Mix I, 1 μL RT Primer Mix and 4 μL RNase Free dH_2_O to the reaction tube; and (d) we incubated the mixture at 37 °C for 15 min, at 85 °C for 5 s before store at −20 °C until use. Table 1 shows gene-specific primers for real-time RT-PCR.

A Mx3005P Real-Time PCR System (Stratagene, Valencia, CA, USA) was used to carry out the mRNA quantification, which was performed in a 10-μL reaction volume, containing 1 μL of cDNA, 5 μL of 2× TB Green Premix Ex Taq (Takara, RR420A), 0.2 μL ROX Reference Dye II, 3.4 μL of RNase-free water and 0.2 μL each of forward and reverse gene-specific primers (10 μM, the total, final concentration). The following cycle amplification conditions were used: (a) an initial denaturation step at 95 °C for 5 min; (b) 40 cycles at 95 °C for 5 s; and (c) 59 °C for 34 s for *IGF1*, 60 °C for 34 s for *BDNF*, 58 °C for 34 s for *TGFB1*, 59 °C for 34 s for *CRHBP*, 63 °C for 34 s for 11-beta hydroxysteroid dehydrogenase 1 (*HSD11B1*) and *HSD11B2*. To determine reaction specificity, PCR products were analyzed by sequencing, dissociation-curve analysis and gel electrophoresis. Gene expression was normalized to internal control (*GAPDH*), and the values were expressed relative to calibrator samples by using the 2^−(ΔΔCT)^ method. For the OECs, each treatment was repeated three times on three different experimental days with each replicate including cells from one well of a 24-well plate, and for embryos, each treatment was repeated three times with each replicate including 250 2- or 4-cell embryos.

We used the Normfinder (https://www.moma.dk/normfinder-software/, accessed on 30 May 2022), an algorithm-based tool, to identify the most stable genes among the six commonly used reference genes in samples derived from 10 different primary pOECs cultures. The ranking of expression stability calculated by Normfinder in the genes analyzed was *GAPDH* = *HMBS* > *ACTB* > *HPRT1* > *RPL13A* > *YWHAZ*, and thus, Gapdh could be used as a stable internal reference gene in our experiments.

We conducted the temperature gradient PCR using the GS00482 Multi Block Thermal Cycler (GSTORM, Somerton, UK). Total RNA was extracted from OECs as described above. Approximately 300 2-cell and 4-cell stage embryos or 50 blastocysts were used to isolate total RNA using a RNAqueous-Micro Total RNA Isolation Kit (AM1931, 202Ambion, Austin, TX, USA). Reverse transcription was conducted as mentioned above. Amplification reactions were performed in a 15 μL reaction volume containing 0.6 μL of cDNA, 1.5 μL of 10× PCR buffer, 0.97 μL dNTP, 0.06 μL Taq (Takara, R006A), 11.27 μL RNase-free water and 0.3 μL each of forward and reverse gene-specific primers (10 μM, Table 1). Amplification started at 95 °C for 3 min followed by 40 cycles at 95 °C for 30 s, gradient temperature 55 °C to 65 °C for 30 s and 72 °C for 30 s; and finally ended with 72 °C for 10 min. We ran the PCR product in 1% agarose gel and isolated desired bands and photographed them under ultraviolet light (Azure c300, Azure biosystems, Dublin, CA, USA). Each treatment was repeated three times with each replicate containing 300 2- or 4-cell embryos or 50 blastocysts.

### 2.10. One-Step PCR for Blastocysts

To extract RNA, 10 blastocysts were lysed with a commercial cell lysis kit (CellAmp Direct Prep Kit for RT-PCR & Protein Analysis, Takara, 3733Q). The RNA obtained was analyzed by real-time PCR using One Step TB Green PrimeScript PLUS RT-PCR Kit (Takara, RR096A) and primers for Bcl2, Bax and Actb (Table 1). We used a 10-μL reaction volume for the amplification (0.4 μL each of forward and reverse primers, 2.2 μL RNase Free d H_2_O, 1 μL template, 5 μL 2× One Step TB Green RT-PCR Buffer, 0.2 μL PrimeScript PLUS RTase Mix, 0.6 μL Takara Ex Taq HS Mix, and 0.2 μL ROX Reference Dye II). We analyzed the relative gene expression using the 2^−∆∆CT^ method following normalization against sus scrofa actin beta (*ACTB*). We conducted mRNA quantification with the Mx3005P real-time PCR instrument (Stratagene, Valencia, CA, USA). Each treatment was repeated three times with each replicate including 10 blastocysts.

### 2.11. Enzyme-Linked Immunosorbent Assay (ELISA)

We carried out ELISA for FASL and TNFα in CM using Porcine Factor Related Apoptosis Ligand (FASL) Elisa kit (BlueGene Co., Shanghai, China) and Porcine tumor necrosis factor α (TNF-α) Elisa kit (Mlbio Co., Shanghai, China), respectively. We centrifuged CM for 10 min at 1000× *g*, and added 100 μL of supernatant or standards in the coated wells. We then added 50 μL of conjugate to each well before incubation for 1 h at 37 °C. After we washed the coated wells with washing solution and dried them with paper towels, we added 50 μL of substrates A and B to each well before incubation for 15 min at 37 °C. Lastly, we terminated the reaction with 50 μL of stop solution, and measured the optical density within 15 min at 450 nm using a plate reader (Infinite50, TECAN, Mannedorf, Switzerland). We calculated the concentrations of FASL or TNFα against respective standard curves. Each treatment was repeated three times on three different experimental days with each replicate containing CM from one well of a 24-well plate.

### 2.12. Immunofluorescence

Immunofluorescence microscopy was performed to localize CRHR1, glucocorticoid receptor (GR), TNFR and FAS in embryos. We removed zona pellucida from the 2-cell, 4-cell and blastocyst embryos with 0.5% pronase in PBS at 37 °C. Then, the embryos were treated as follows: (a) fixation for 30 min in 4% paraformaldehyde in PHEM buffer (60 mM Pipes, 25 mM Hepes, 10 mM EGTA and 4 mM MgSO_4_, pH 7); (b) permeabilization with 0.25% TritonX-100 to observe GR; (c) block for 1 h in PBS with 3% bovine serum albumin (BSA); (d) overnight incubation at 4 °C with primary antibodies in PBS with 3% BSA; (e) 1-h incubation with secondary antibodies in PBS with 3% BSA; (f) 10-min incubation in PBS with 10 μg/mL of Hoechst 33342; and (g) observation under a Leica laser-scanning confocal microscope (TCS SP2; Leica Microsystems, GmbH, Wetzlar, Germany). We washed the embryos in PBS between treatments. We detected fluorescence using band pass emission filters (Hoechst 33342, 420–480 nm; Cy3, 560–605 nm). We also processed negative control samples with the primary antibody omitted to verify the specificity of the secondary antibodies.

The primary antibodies used are as follows: goat anti-CRHR1 polyclonal antibodies (1:500, ab59023; Abcam Co., Ltd., Cambridge, MA, USA) for CRHR1; rabbit anti-GR polyclonal antibodies (1:1000, ab196944; Abcam Co., Ltd.) for GR; rabbit anti-TNFR1 polyclonal antibodies (1:200, AF8196, Beyotime Co., Ltd.) for TNFR; and rabbit anti-FAS (1:100, ab82419, Abcam Co., Ltd.) for FAS. Cy3-conjugated AffiniPure goat anti-rabbit IgG (1:500; Jackson ImmunoResearch, West Grove, PA, USA) were used as secondary antibodies for GR, TNFR1 and FAS and Cy3-conjugated donkey anti-goat IgG (1:500; Beyotime Co., Ltd.) were used for CRHR1 secondary antibodies.

### 2.13. RNA Interference

The RiboBio (Guangzhou, China) designed and synthesized the siRNAs targeting mRNAs and the negative control siRNA. The sense strands of siRNAs targeting the *FASL* gene were FL siRNA-1 (5-CAA TCT ACC CTC TGA GAAA-3), FL siRNA-2 (5-GTC AGT ACT GCA ACA ACCA-3), and FL siRNA-3 (5-GAT GAA CTA TTG CAC TACT-3); those targeting the *TNFα* gene were TN siRNA-1 (5-CTC AGA TCA TCG TCT CAAA-3), TN siRNA-2 (5-CGA CTA TCT GGA CTT TGCT-3) and TN siRNA-3 (5-CCT ACC AGA CCA AGG TCAA-3); and we used siR-RiboTM for negative control. We performed transfection with 100 nM siRNAs using the lipofectamine RNAiMAX reagent (Invitrogen/Life Technologies, Grand Island, NY, USA). When OECs grew to approximately 60% of confluence, we replaced the spent medium in the wells with 450 μL fresh DMEM/F-12 medium and transfected the cells using the forward transfection method. To prepare the transfection complex, we diluted 1 μL of a 20 μM solution of each siRNA in 50-μL Opti-MEM medium (Invitrogen), and mixed it with 3 μL of Lipofectamine RNAiMAX reagent (Invitrogen) diluted in 50 μL Opti-MEM medium. Following incubation for 5 min at room temperature, we added 50 μL of the transfection complex to the wells before incubation for 6 h at 38.5 °C in a humidified 5% CO_2_ atmosphere. The transfected cells were further cultured for 48 h in serum-free DMEM/F12 with or without CRH or cortisol before recovery of CM or cells for ELISA measurement of FASL or TNFα or for flow cytometry analysis of apoptosis, respectively.

### 2.14. Data Analysis

Each treatment contained at least three replicates, unless otherwise specified. We used the software of Statistics Package for Social Sciences (SPSS 11.5, IBM, Chicago, IL, USA) to perform data analysis. We arc sine-transformed the percentage data before analysis. We used independent *t*-test to analyze data when each measure contained only two groups, and used ANOVA when each measure had more than two groups. We carried out a Duncan multiple comparison test to locate differences during ANOVA. We expressed data as means ± SEM, and considered differences significant when *p* < 0.05.

## 3. Results

### 3.1. Effects of Treatment with CRH, ACTH or Cortisol on In Vitro Development of Pig Embryos

**Figure 1:**
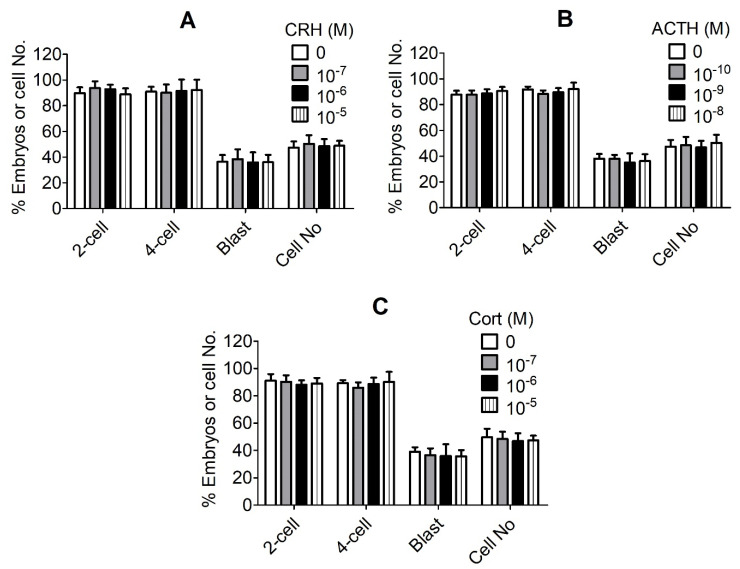
Effects of culture with different concentrations of corticotrophin-releasing hormone (CRH), adrenocorticotropic hormone (ACTH) or cortisol (Cort) on the embryo development of pig activated oocytes. Graphs (**A**–**C**) show embryo development after culture with CRH, ACTH and Cort, respectively. Matured (MII) oocytes showing first polar body (PB1) were chemically activated and cultured in the presence of different concentrations of CRH, ACTH or Cort before observation for embryo development. Three concentrations of each hormone, plus a blank control, were used. Each treatment was repeated four times with each replicate containing approximately 25 MII oocytes. While 2-cell and 4-cell embryos were observed at 48 h, blastocysts were observed on day 6 after the onset of embryo culture. Percentages of 2-cell, 4-cell and blastocyst (Blast) embryos were calculated from MII oocytes, 2-cell and 4-cell embryos, respectively. Cell numbers (Cell No) per blastocyst were counted from all the blastocysts collected from each treatment. No significant effect of any concentration of any hormone was observed on any stage of embryo development.

In vitro matured (MII) pig oocytes showing the first polar body (PB1) were chemically activated and cultured in the presence of different concentrations of CRH, ACTH or cortisol for 7 days before examination for embryo development. The results revealed no significant effects of any concentration of any hormone tested on any stage of embryo development (Figure 1). Because it is known that the stress-induced serum concentrations of CRH [20], ACTH [21] and cortisol [22] in pigs are 1 × 10^−8^ M, 3 × 10^−11^ M and 4 × 10^−7^ M, respectively, our results suggest that none of the three hormones impaired development of pig preimplantation embryos even at levels much higher than the physiological concentrations.

### 3.2. Effects of Treatment with CRH, ACTH or Cortisol on Apoptosis of OECs

**Figure 2:**
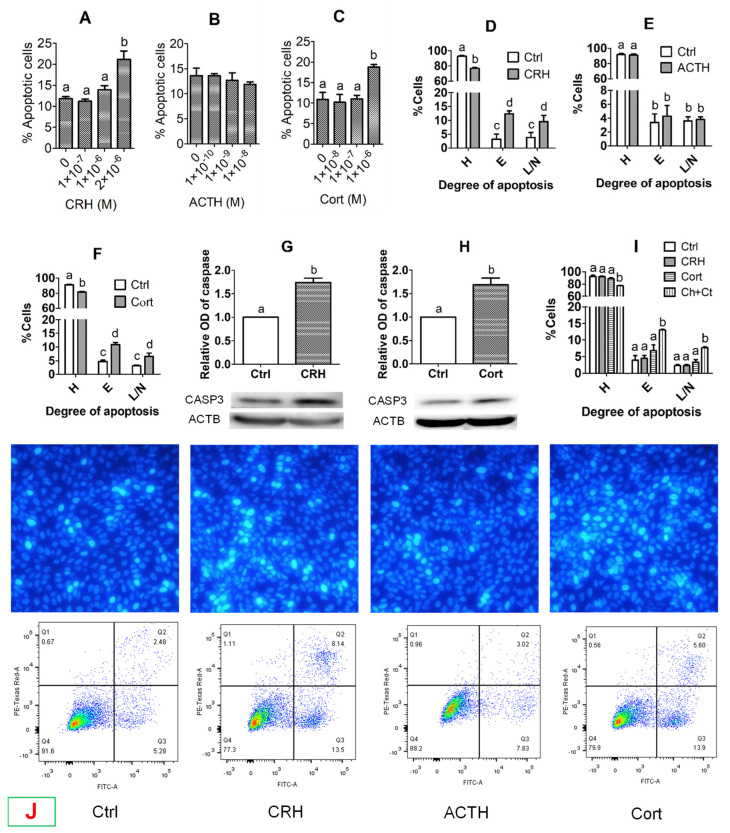
Effects of treatment with corticotrophin-releasing hormone (CRH), adrenocorticotropic hormone (ACTH) or cortisol (Cort) on the apoptosis of pig oviduct epithelial cells (OECs). While graphs (**A**–**C**) show Hoechst staining-revealed percentages of apoptotic cells following culture of OECs with different concentrations of CRH, ACTH or Cort, respectively, graphs (**D**–**F**) show flow cytometry-revealed percentages of healthy (**H**), early apoptotic (**E**) and late apoptotic/necrotic (L/N) cells, following the culture of OECs with CRH (2 × 10^−6^ M), ACTH (1 × 10^−8^ M) or Cort (1 × 10^−6^ M), respectively. Graphs (**G**,**H**) show relative levels of active caspase 3 (CASP3) as revealed by Western blotting after the OECs were cultured with CRH (2 × 10^−6^ M) and Cort (1 × 10^−6^ M), respectively. Graph (**I**) shows flow cytometry-revealed percentages of healthy (**H**), early apoptotic (**E**) and late apoptotic/necrotic (L/N) cells, following the culture of OECs without hormone (Ctrl) or with CRH (1 × 10^−8^ M) or Cort (4 × 10^−7^ M) alone or both (Ch + Ct). Each treatment was repeated three times with each replicate including cells from one well of a 12-well culture plate. a–d: Values with a different letter above bars differ (*p* < 0.05). Panel (**J**) shows micrographs after Hoechst 33342 staining (upper row) and the flow cytometry graphs (lower row) following OECs were cultured with CRH (2 × 10^−6^ M), ACTH (1 × 10^−8^ M) or Cort (1 × 10^−6^ M), respectively. Control (Ctrl) OECs were cultured without stress hormone. In the Hoechst-staining micrographs, the apoptotic cells with pyknotic nuclei were heavily stained by Hoechst 33342 emitting bright light. The micrographs were taken at an original magnification of ×400. In the flow cytometry graphs, the healthy, early apoptotic and late apoptotic/necrotic cells are located in the Q4, Q3 and Q2 areas, respectively. The Q1 area contains mechanically damaged cell debris.

When OECs grew to approximately 80% of confluence, they were cultured with different concentrations of CRH, ACTH or cortisol for 48 h before examination for apoptosis. Our Hoechst staining showed that the percentages of apoptotic cells increased significantly when concentrations of CRH and cortisol increased to 2 × 10^−6^ M and 1 × 10^−6^ M, respectively (Figure 2A,C,J). However, no significant change in the percentage of apoptotic cells was observed when OECs were cultured with high concentrations of ACTH (Figure 2B,J). Our flow cytometry indicated that while the percentages of healthy cells decreased, the percentages of early and late apoptotic/necrotic cells increased significantly after treatment of OECs with 2 × 10^−6^ M of CRH or 1 × 10^−6^ M of cortisol (Figure 2D,F,J). Treatment with ACTH, however, changed neither the percentages of healthy nor apoptotic cells significantly (Figure 2E,J). Our Western blotting further confirmed that treatment of OECs with 2 × 10^−6^ M of CRH or 1 × 10^−6^ M of cortisol significantly increased their levels of active caspase-3 (Figure 2G,H). Taken together, the results demonstrated that treatment with CRH or cortisol dose-dependently induced significant apoptosis of pig OECs, whereas treatment with ACTH showed no effect.

However, the effective concentrations of CRH and cortisol that caused significant apoptosis of pig OECs were higher than peak serum concentrations reported in stressed pigs. Our hypothesis was that in vivo, stresses increase the release of multiple hormones that work together to induce a symptom at lower concentrations than in vitro in the presence of only a single hormone. To test this hypothesis, pig OECs were cultured for 48 h with stress-induced concentration of CRH (1 × 10^−8^ M) or cortisol (4 × 10^−7^ M) alone or both before examination for apoptosis by flow cytometry. The results show that although culture with either 1 × 10^−8^ M CRH or 4 × 10^−7^ M cortisol alone did not increase apoptosis, culture with both 1 × 10^−8^ M CRH and 4 × 10^−7^ M cortisol significantly increased early and late apoptosis of pig OECs (Figure 2I). The results suggest that in vivo, multiple hormones may work together to induce cell apoptosis at lower concentrations.

### 3.3. Effects of Treatment with CRH, ACTH or Cortisol on In Vitro Development of Pig Embryos Cocultured with OECs

**Figure 3:**
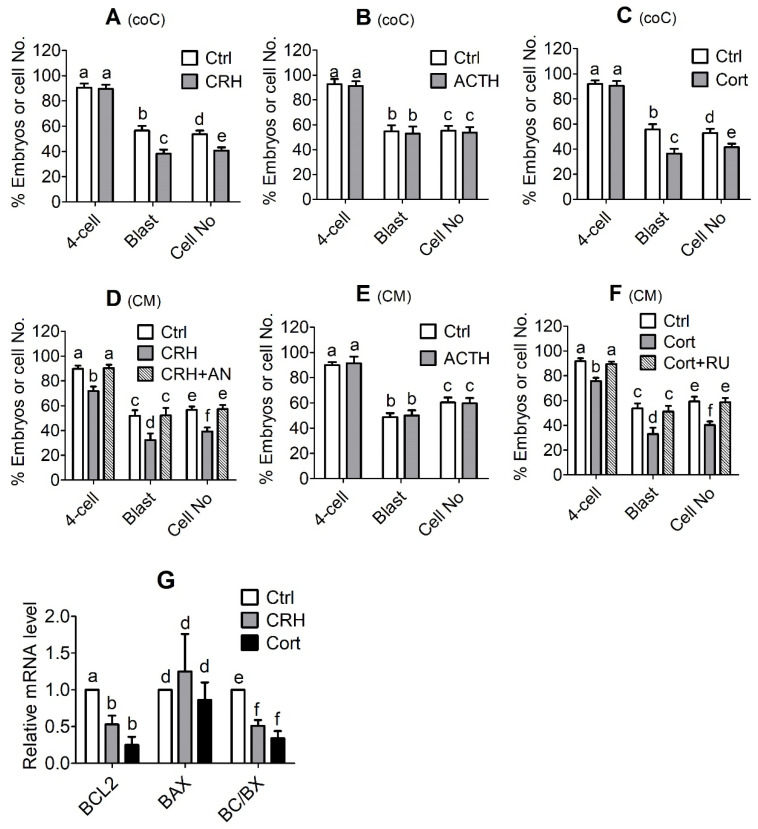
In vitro development and apoptosis of pig embryos cocultured with oviduct epithelial cells (OECs) in the presence of corticotrophin-releasing hormone (CRH), adrenocorticotropic hormone (ACTH) or cortisol (Cort) or cultured in medium conditioned by OECs pretreated with CRH, ACTH or cortisol. In graphs (**A**–**C**), MII oocytes showing first polar body (PB1) were chemically activated and cocultured (coC) with OECs in the presence of CRH (2 × 10^−6^ M), ACTH (1 × 10^−8^ M) or Cort (1 × 10^−6^ M), respectively, before observation for embryo development. Control (Ctrl) oocytes were cocultured with OECs without stress hormones. In graphs (**D**–**G**), activated oocytes were cultured in conditioned medium (CM) before examination for embryo development or apoptosis, and the CM was conditioned for 24 h with OECs that had been pretreated for 48 h with CRH (2 × 10^−6^ M), ACTH (1 × 10^−8^ M) or Cort (1 × 10^−6^ M), respectively. Control CM was conditioned with OECs pretreated without stress hormones. CRH + AN: OECs were pretreated for 48 h with both CRH and antalarmin (2 × 10^−6^ M); Cort + RU: OECs were pretreated for 48 h with both Cort and RU486 (1 × 10^−6^ M). While 2-cell and 4-cell embryos were observed at 48 h, blastocysts were observed on day 6 after the onset of embryo culture. Percentages of 2-cell, 4-cell and blastocyst (Blast) embryos were calculated from MII oocytes, 2-cell and 4-cell embryos, respectively. Graph G shows mRNA levels of *BCL2* and *BAX* and the ratio of *BCL2/BAX* (BC/BX) as measured by one step RT-PCR. a–f: Values with a different letter above the bars differ significantly (*p* < 0.05).

MII oocytes showing PB1 were chemically activated and cocultured with OECs in the presence of CRH (2 × 10^−6^ M), ACTH (1 × 10^−8^ M) or cortisol (1 × 10^−6^ M) for 7 days before observation for embryo development. Control oocytes were cocultured with OECs without stress hormones. Although culture with ACTH showed no effect, the presence of CRH or cortisol significantly decreased the blastocyst rates and the cell number per blastocyst in pig embryos cocultured with OECs (Figure 3A–C). The results suggest that CRH and cortisol impaired pig embryo development indirectly through actions on OECs.

### 3.4. In Vitro Development and Apoptosis of Pig Embryos Cultured in CM Conditioned with CRH-, ACTH- or Cortisol-Pretreated OECs

The activation-treated MII oocytes were cultured for 7 days in CM conditioned with CRH-, ACTH- or cortisol-pretreated OECs before observation for embryo development or apoptosis. The control embryos were cultured in CM conditioned with OECs that were pretreated without stress hormones. Although CM conditioned with ACTH-pretreated OECs had no effect, CM conditioned by CRH- or cortisol-pretreated OECs significantly decreased the blastocyst rates and cell number per blastocyst of pig embryos (Figure 3D–F). When the pretreatment of OECs involved CRH or glucocorticoid receptor antagonists (antalarmin or RU486), the detrimental effects of CRH or cortisol CM on embryo development disappeared completely, suggesting that (a) CRH and cortisol altered the function of OECs through the interaction with their respective receptors, and (b) the two hormones were nontoxic when used at the current concentrations because when OECs used for CM preparation were pretreated with both the antagonists and CRH/cortisol, blastocyst rates and cell number per blastocyst were at the same levels as those when control CM was prepared with OECs precultured without CRH/cortisol. Furthermore, culture with CM conditioned by CRH- or cortisol-pretreated OECs significantly decreased the ratio of Bcl-2/Bax mRNAs in blastocysts (Figure 3G). Because the above results showed that when used in combination, stress-induced concentrations of CRH and cortisol induced significant apoptosis of pig OECs, the results suggest that CRH and cortisol might impair development and cause apoptosis of pig embryos in vivo indirectly by acting on OECs.

### 3.5. Fertilization in CM Conditioned with CRH- or Cortisol-Pretreated OECs Increased Rates of Polyspermy

**Figure 4:**
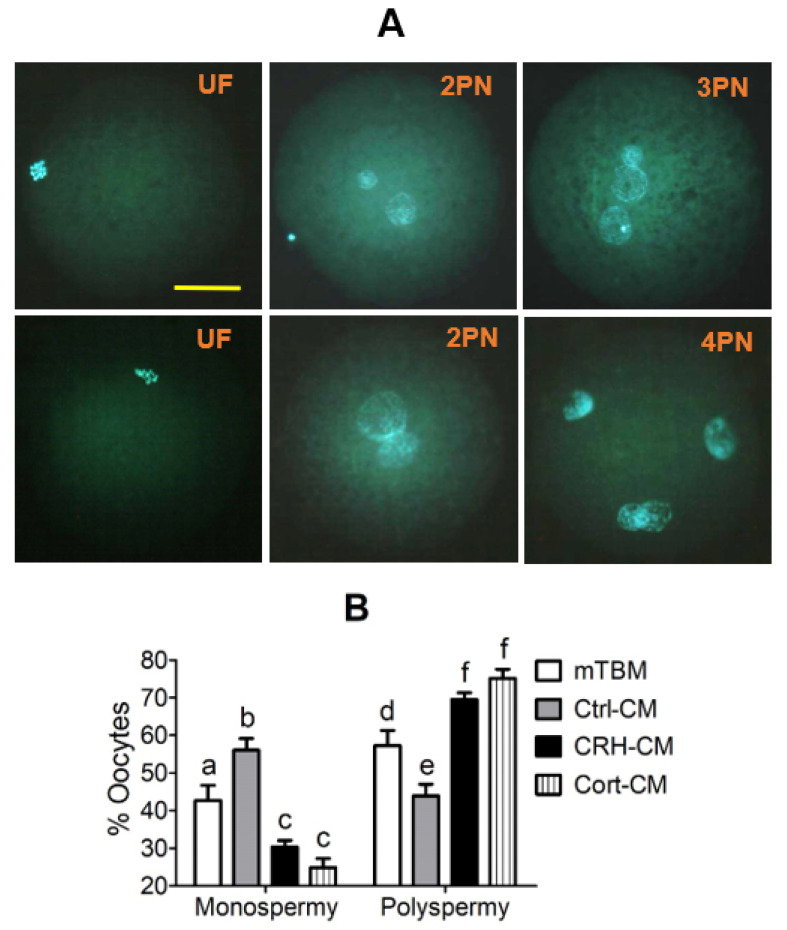
Fertilization of pig oocytes in modified Tris Buffer Media (mTBM)-Conditioning Media (mTBM-CM) conditioned with corticotrophin-releasing hormone (CRH)- or cortisol (Cort)-pretreated oviduct epithelial cells (OECs). Panel (**A**) shows micrographs of unfertilized (UF) oocytes, and fertilized oocytes with two (2PN), three (3PN) or four pronuclei (4PN) as observed under a fluorescence microscope at a magnification of ×400 following Hoechst 33342 staining. The bar is 32 μm and applies to all images. Graph (**B**) shows percentages of oocytes with monospermy or polyspermy after insemination of oocytes in mTBM alone, or in mTBM-CM conditioned with OECs without hormone pretreatment (Ctrl-CM), or with OECs pretreated with either CRH (CRH-CM) or cortisol (Cort-CM). a–f: Values with a different letter above the bars differ significantly (*p* < 0.05).

MII oocytes showing PB1 were inseminated with fresh semen in mTBM alone, or in mTBM-CM conditioned with OECs without hormone pretreatment (Ctrl-CM), or with OECs pretreated with either CRH (CRH-CM) or cortisol (Cort-CM) before examination for fertilization. Oocytes with one or no pronucleus, oocytes with two pronuclei and those with three or more pronuclei were considered unfertilized, monospermic and polyspermic, respectively (Figure 4A). Although total fertilization rates (ranging from 75.3 ± 2.6 % to 82.1 ± 8.9%) did not differ among treatments, percentages of polyspermic oocytes were significantly higher when fertilization took place in CRH-CM or Cort-CM than in Ctrl-CM (Figure 4B). The polyspermic rates in oocytes inseminated in mTBM alone were significantly higher than those inseminated in Ctrl-CM but significantly lower than those inseminated in CRH-CM or Cort-CM. The results suggest that while CM conditioned by healthy OECs inhibited polyspermy, CM conditioned by apoptotic OECs facilitated polyspermy.

### 3.6. Effects of Treatment with CRH or Cortisol on Expression of Growth Factors and Oviduct-Specific Glycoprotein (OVGP1) in OECs

**Figure 5:**
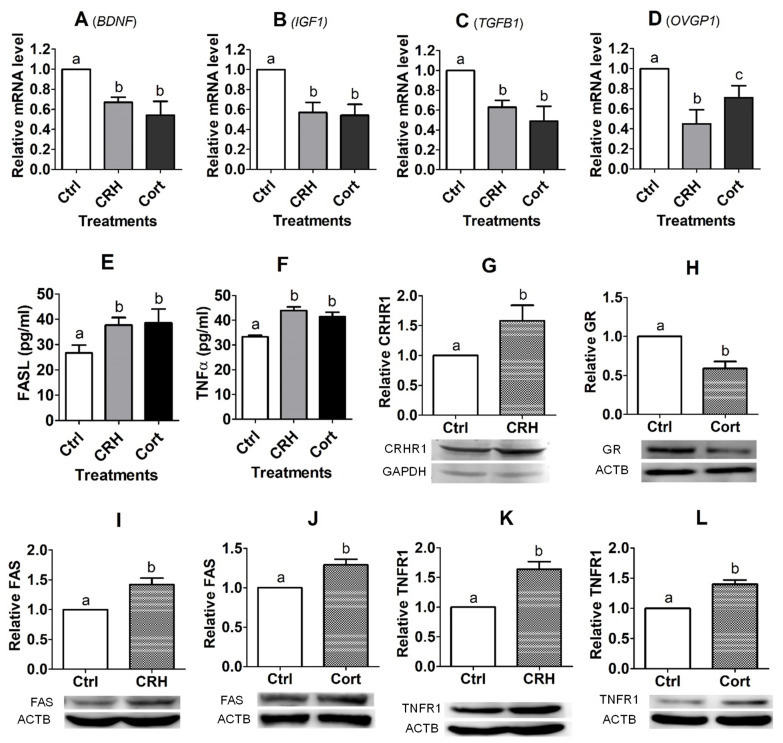
Effects of culture with corticotrophin-releasing hormone (CRH) or cortisol (Cort) on expression of growth factors, oviduct-specific glycoprotein (OVGP1), Fas ligand (FASL)/tumor necrosis factor (TNF) α and their receptors, and CRH/glucocorticoid receptors (GR) in pig oviduct epithelial cells (OECs). Pig OECs were cultured without (Ctrl) or with CRH (2 × 10^−6^ M) or Cort (1 × 10^−6^ M) for 48 h before examination. Graphs (**A**–**D**) show relative levels (RT-PCR results) of brain-derived neurotropic factor (*BDNF)*, insulin-like growth factor 1 (*IGF1)*, transforming growth factor β1 (*TGFB1*) and *OVGP1* mRNAs, respectively. Graphs (**E**,**F**) show levels of FASL and TNFα proteins (ELISA results), respectively. Graphs (**G**–**L**) show relative levels of CRH receptor (CRHR1), GR, FAS and TNFR1, respectively, as revealed by Western blotting. a–c: Values with a different letter above bars differ significantly (*p* < 0.05).

Upon growing to approximately 80% of confluence, OECs were cultured with 2 × 10^−6^ M of CRH or 1 × 10^−6^ M of cortisol for 48 h before RT-PCR assays. Treatment with either CRH or cortisol significantly decreased the expression of *BDNF*, *IGF1*, *TGFB1* and *OVGP1* mRNAs (Figure 5A–D). The level of *OVGP1* mRNA was higher significantly following the cortisol than CRH treatment (Figure 5D), suggesting that CRH inhibited the *OVGP1* expression of pig OECs more severely than cortisol did. Thus, the results suggest that treatment with CRH or cortisol significantly suppressed the expression of growth factors and OVGP1 in pig OECs.

### 3.7. Effects of CRH or Cortisol Treatment on Secretion of FASL and TNFα in OECs

Pig OECs were treated with 2 × 10^−6^ M of CRH or 1 × 10^−6^ M of cortisol for 48 h before recovery of CM for ELISA measurement for FASL and TNFα concentrations. The results showed that the treatment of pig OECs with either CRH or cortisol significantly enhanced the expression of proapoptotic factors FASL and TNFα (Figure 5E,F). Because it was shown that culture of mouse OECs with corticosterone decreased TNFα expression significantly [23], contrary to the present results with pig OECs, we measured the TNFα level in CM from both pig and mouse OECs using the same ELISA kit. The results showed that while the TNFα concentration in CM from cortisol-treated pig OECs (40.09 ± 2.35 pg/mL) was significantly (*p* < 0.05) higher than that in CM from the untreated control OECs (27.83±2.66 pg/mL), the TNFα concentration in CM from corticosterone-treated mouse OECs (33.00 ± 6.15 pg/mL) was significantly (*p* < 0.05) lower than that in CM from control OECs (69.59 ± 7.10 pg/mL). The results confirmed the species difference in the effect of glucocorticoids on TNFα expression in OECs.

### 3.8. Effects of CRH or Cortisol Treatment on Expression of CRHR, GR, FAS and TNFR1 in OECs

Pig OECs were treated with 2 × 10^−6^ M of CRH or 1 × 10^−6^ M of cortisol for 48 h before the Western blotting analysis. While treatment with CRH significantly increased CRHR1 expression, treatment with cortisol decreased GR expression significantly (Figure 5G,H). Treatment with either CRH or cortisol significantly increased the expression of FAS and TNFR1 (Figure 5I–L). The results suggest that treatment with CRH or cortisol enhanced interactions with their respective receptors, activating apoptosis pathways of Fas and TNF-α signaling.

### 3.9. Effects of Knocking down FASL or TNFα on Apoptosis of CRH- or Cortisol-Treated OECs

**Figure 6:**
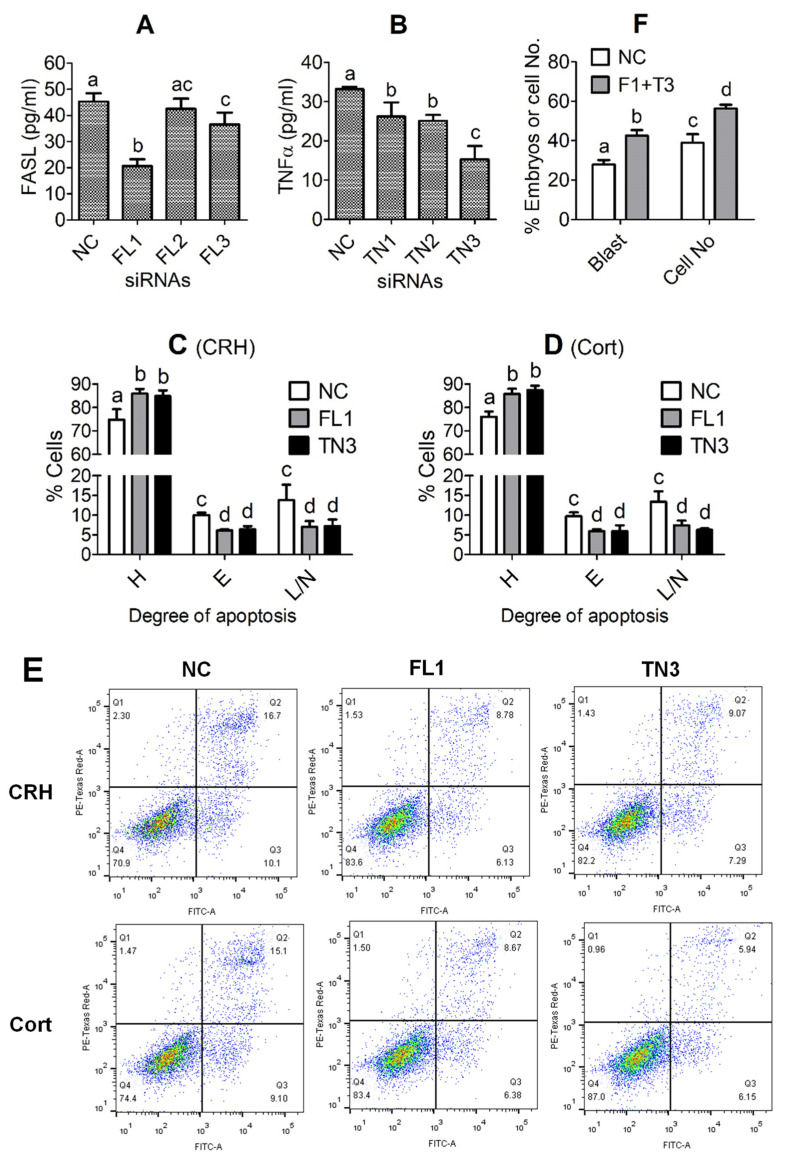
Effects of knocking down Fas ligand (*FASL*) or tumor necrosis factor (*TNFα*) on corticotrophin-releasing hormone (CRH)- or cortisol (Cort)-induced apoptosis of oviduct epithelial cells (OECs). Graphs (**A**,**B**) show FASL and TNFα concentration, respectively, in CM conditioned for 48 h without hormones with OECs transfected with negative control (NC), *FASL* siRNA-1 (FL1), -2 (FL2) or -3 (FL3), or with *TNFα* siRNA-1 (TN1), -2 (TN2) or -3 (TN3). Graphs (**C**,**D**) show the percentages of healthy (H), early apoptotic (E) or late apoptotic and necrotic (L/N) cells as revealed by flow cytometry following OECs transfected with NC, FL1 or TN3 siRNAs were cultured for 48 h in the presence of CRH and Cort, respectively. Panel (**E**) shows the flow cytometry graphs after the OECs transfected with NC, FL1 or TN3 siRNAs were cultured with CRH or Cort for 48 h. The healthy, early apoptotic and late apoptotic/necrotic cells are located in the Q4, Q3 and Q2 areas, respectively. The Q1 area contains mechanically damaged cell debris. Graph (**F**) shows the percentages of blastocysts (Blast) and cell number per blastocyst (Cell No) following embryo culture in CM conditioned in the presence of both 1 × 10^−8^ M CRH and 4 × 10^−7^ M cortisol with OECs that had been transfected with NC siRNA or co-transfected with both *FASL* siRNA-1 and *TNFα* siRNA-3 (F1 + T3). a–d: Values with a different letter above the bars differ significantly (*p* < 0.05).

To test the efficiency of different siRNA sequences, the OECs that had been transfected with negative control (NC), FASL or TNFα siRNAs were cultured in DMEM/F12 without hormones for 48 h before recovery of CM for ELISA measurement for FASL or TNFα. The FASL and TNFα levels decreased significantly after transfection with *FASL* siRNA-1 and *TNFα* siRNA-3, respectively, compared to transfection with NC siRNA (Figure 6A,B). To study the effects of silencing *FASL* or *TNFα* on CRH- or cortisol-induced apoptosis, the OECs were transfected with NC siRNA, *FASL* siRNA-1 or *TNFα* siRNA-3, and then cultured in DMEM/F12 containing CRH or cortisol for 48 h before apoptosis assessment using flow cytometry. The percentages of both early apoptotic and late apoptotic or necrotic cells decreased significantly after transfection with *FASL* siRNA-1 or *TNFα* siRNA-3, compared to those after transfection with NC siRNA, whether cultured in the presence of CRH (Figure 6C,E) or cortisol (Figure 6D,E). The results indicated that silencing either *FASL* or *TNFα* significantly alleviated the proapoptotic effects of CRH or cortisol on pig OECs, suggesting that both CRH- and cortisol-induced pig OEC apoptosis involved both FAS signaling and TNFα signaling.

To further reinforce the indirect effects of the stress hormone-induced OECs apoptosis on preimplantation embryo development, pig parthenotes were cultured in CM conditioned in the presence of both 1 × 10^−8^ M CRH and 4 × 10^−7^ M cortisol with OECs that had been transfected with NC siRNA or co-transfected with both *FASL* siRNA-1 and *TNFα* siRNA-3. Co-transfection of OECs with *FASL* and *TNFα* siRNAs significantly (*p* < 0.05) increased both the blastocyst rates and the cell number per blastocyst in comparison with those following transfection with NC siRNA (Figure 6F). The results further substantiated that the stress hormone-induced OECs apoptosis impaired embryo development by releasing more FASL and TNFα.

### 3.10. Expression of CRHR1, GR, FAS, and TNFR1 in Preimplantation Embryos at Different Developmental Stages

**Figure 7:**
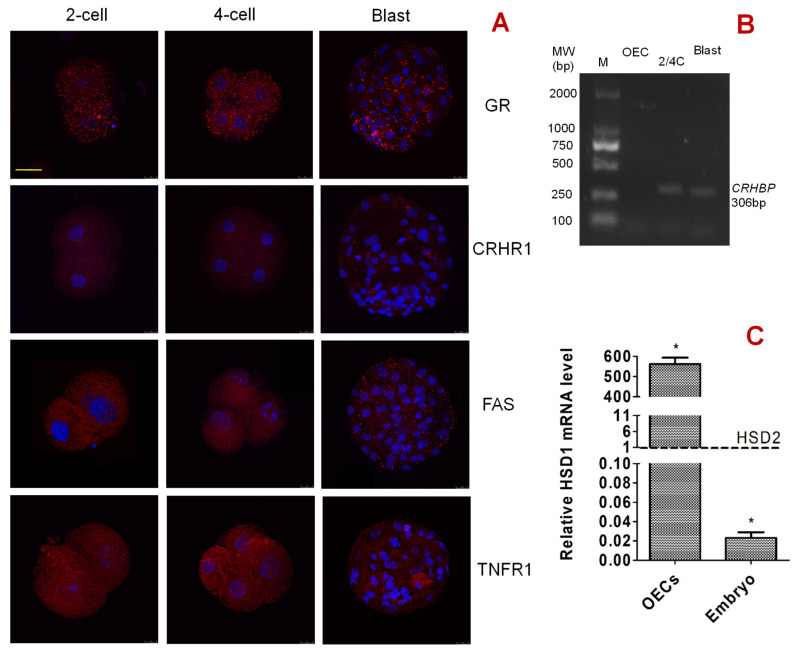
Expression of corticotrophin-releasing hormone receptor (CRHR1), glucocorticoid receptor (GR), FAS, tumor necrosis factor receptor (TNFR1), corticotrophin-releasing hormone-binding protein (CRHBP) and hydroxysteroid 11-beta dehydrogenase 1/2 (HSD11B1/HSD11B2) in pig preimplantation embryos. Panel (**A**) shows expression of CRHR1, GR, FAS, and TNFR1 in 2-cell, 4-cell and blastocyst (Blast) embryos, as revealed by immunofluorescence microscopy. The micrographs are merged images from laser confocal microscopy with target proteins and DNA colored red and blue, respectively. The bar is 50 μm and applies to all images. While 2-cell and 4-cell embryos were observed at 48 h, the blastocysts were observed on day 6 after the onset of embryo culture. Panel (**B**) shows mRNA expression of *CRHBP* in pig oviduct epithelial cells (OECs), 2- or 4-cell embryos (2/4C) and blastocysts (Blast), as revealed by a general PCR amplification. The annealing temperature for the general PCR amplification was set to 58 °C. M: DM 2000 DNA marker. The molecular weight for *CRHBP* mRNA is 306 bp. Graph (**C**) shows relative levels of *HSD11B1* (HSD1) and *HSD11B2* (HSD2) mRNAs in pig embryos and OECs. The level of *HSD11B2* mRNA was set as one (dotted line), and the level of *HSD11B1* mRNA was expressed relative to it. * Indicates significant difference from the value of *HSD11B2*.

Activation-treated oocytes were cultured in PZM-3 medium alone before observation by immunofluorescence microscopy. While 2-cell and 4-cell embryos were observed at 48 h, blastocysts were observed on day 6 after the onset of embryo culture. Our immunofluorescence microscopy demonstrated that GR, FAS and TNFR1 were expressed in pig embryos at the 2-cell, 4-cell and blastocyst stages, and they were localized mainly in the cytoplasm and plasma membrane of blastomeres (Figure 7A). However, CRHR1 was not expressed until the blastocyst stage, when only a few puncta were observed in the cytoplasm and plasma membrane.

### 3.11. Expression of CRH-Binding Protein (CRHBP) and HSD11B1/HSD11B2 in Pig Embryos and OECs

Activation-treated oocytes were cultured in PZM-3 medium alone for 48 h and 6 days to recover 2-/4-cell embryos and blastocysts, respectively. Upon growing to approximately 80% of confluence, OECs were cultured in DMEM-F12 medium alone for 48 h before recovery for analysis. Our general PCR amplification indicated that *CRHBP* mRNA was expressed in pig 2- and 4-cell embryos and blastocysts but not in the OECs (Figure 7B), suggesting that the expression of CRHBP is one of the reasons for pig embryos’ tolerance to CRH treatment. Our RT-PCR showed that in pig 2- and 4-cell embryos, the mRNA level of *HSD11B2* was 44.8 times as much as that of *HSD11B1* (Figure 7C). In OECs, however, the mRNA level of *HSD11B1* was 562.2 times as much as that of *HSD11B2*. Because it is known that HSD11B1 is a reductase to generate active cortisol or corticosterone, and HSD11B2 catalyzes the conversion of glucocorticoids to inactive cortisone [24], the results suggest that pig embryos are insensitive to cortisol because they contained much more HSD11B2 than HSD11B1.

## 4. Discussion

Although it was reported that repeated ACTH stimulation [16] or food deprivation [20] after ovulation in sows significantly impaired the cleavage of the preimplantation embryos while increasing the level of serum cortisol, the mechanisms by which the stress hormones affect embryo development are largely unknown. In this study, we first observed that the preimplantation development of pig embryos was unaffected when the parthenotes were cultured with various concentrations of CRH, ACTH or cortisol, ruling out the possibility that these stress hormones affect the pig embryos directly. However, Razdan et al. [16] observed that the stimulation of sows with ACTH impaired the cleavage of the preimplantation embryos in vivo. The present results suggest that in the study by Razdan et al. [16], it might be the ACTH-induced elevation of cortisol that triggered OECs apoptosis and impaired embryo cleavage. To find out why CRH and cortisol did not affect the pig embryos directly, we found that pig embryos did not express CRHR1 until the blastocyst stage, and that they expressed CRHBP at all stages of the preimplantation development. It is known that CRHBP can bind CRH with an affinity greater than the CRHR to modulate the CRHR activity [25], and it inhibits the CRH-induced ACTH secretion from pituitary corticotropes [26]. Although pig embryos expressed GR at all the preimplantation stages observed, their mRNA level of *HSD11B2* was 45 times as much as that of *HSD11B1*. According to Michael et al. [24], whereas HSD11B1 can activate (reduce) cortisone to cortisol or corticosterone, and HSD11B2 can inactivate (oxidize) cortisol or corticosterone to cortisone. Michael et al. [27] observed that human female conception rates following in vitro fertilization were associated with an increased ratio of cortisol:cortisone in follicular fluid, due to a low level of cortisol oxidation by HSD11B2. Furthermore, Gong et al. [28] reported that the species difference in glucocorticoid sensitivity between pig and mouse oocytes can be attributed to their different contents/ratios of HSD11B1 and HSD11B2, which maintain different concentrations of active glucocorticoids.

However, preimplantation development was significantly impaired when the pig parthenotes were cocultured with the OECs in the presence of CRH or cortisol or when they were cultured in CM conditioned with CRH- or cortisol-pretreated OECs. Furthermore, fertilization in CM conditioned with CRH- or cortisol-pretreated OECs significantly increased rates of polyspermy. Further investigation indicated that pig OECs underwent significant apoptosis and produced significantly less growth factors and OVGP1 after culture with CRH or cortisol. In addition, pig OECs also produced more FASL/TNFα when cultured in the presence of CRH or cortisol, and embryo culture in CM conditioned by OECs co-transfected with *FASL* and *TNFα* siRNAs significantly increased blastocyst rates and the cell number per blastocyst, compared with those following transfection with NC siRNA. Our immunofluorescence microscopy confirmed that FAS and TNFR1 were expressed in pig embryos at the 2-cell, 4-cell and blastocyst stages. There are many reports that OVGP1 secreted by OECs around ovulation time causes pre-fertilization zona hardening which contributes to polyspermy block in mammals, including pigs [29,30]. Furthermore, it has been reported that a higher incidence of polyspermy was associated with a lower level of plasma IGF1 in sows with a lactational negative energy balance [31]. Taken together, the current results suggest that CRH and cortisol impaired fertilization and the development of pig preimplantation embryos by inducing the apoptosis of OECs and the apoptotic OECs damaged embryos by producing less antiapoptotic growth factors and OVGP1 but more proapoptotic FASL and TNFα. Because our previous study, using mouse zygotes, obtained similar results that CRH/cortisol impaired development of fertilized embryos indirectly by inducing OECs apoptosis [8], the present results suggest that parthenotes might have the same sensitivity to CRH/cortisol as fertilized embryos do.

Many papers have documented that CRH or cortisol trigger apoptosis in various somatic cells. For instance, CRH has been reported inducing apoptosis in neurons [32], in PC12 rat pheochromocytoma cell line [33], in microglial cells [34], in prostate cancer cell RM-1 [35] and in the mural granulosa cells (MGCs) of mouse ovarian follicles [36,37]. It is widely known that glucocorticoids can cause apoptosis in T lymphocytes [38]. Treatment of mice with dexamethasone significantly increased apoptosis in testicular germ cells [39]. Culture with dexamethasone induced apoptosis in monocytes [40] and osteocytes [41]. Furthermore, injection of mice with cortisol [42] or culture with corticosterone [43] induced apoptosis in mouse MGCs.

The present results show that treatment with either CRH or cortisol significantly increased the expression of both FASL and TNFα and their receptors in pig OECs. Furthermore, knocking down either *FASL* or *TNFα* by RNA interference significantly reduced the apoptotic percentages of pig OECs following culture in the presence of either CRH or cortisol. Taken together, the results suggest that both CRH and cortisol induced the apoptosis of pig OECs by activating both the FASL/FAS system and the TNFα/TNFR1 signaling. Both in vitro and in vivo studies have demonstrated that CRH increased expression of FASL and/or FAS in various somatic cells [36,44,45,46,47], and silencing the *FASL* gene by RNA interference significantly alleviated the proapoptotic effect of CRH on mouse MGCs [36]. Song et al. [48] reported that CRH promoted TNFα production by CD14+ cells. Zhao et al. [37] observed that CRH treatment of mouse MGCs significantly increased their apoptotic percentages and levels of TNFα and TNFR1 expression, and in vitro knockdown by interfering RNA or in vivo knockout of the *TNFα* gene significantly alleviated the proapoptotic effect of CRH on MGCs.

Culture with glucocorticoids activated the Fas/FasL system in osteocytes and monocytes [40,41]. Treatment of mice with dexamethasone significantly increased FASL expression in testicular germ cells [39]. The injection of mice with cortisol increased FASL secretion in ovaries and FAS expression in MGCs, cumulus cells (CCs) and oocytes, and the proapoptotic effect of cortisol injection on MGCs and CCs were significantly relieved when the *gld* mice harboring *FASL* mutations were observed [42]. While Dinkel et al. [49] reported that glucocorticoids upregulated the *TNFα* signaling in rat CNS, Messmer et al. [50] and Zhang et al. [51] documented that the glucocorticoids inhibited TNFα expression in human mammary carcinoma cell line MCF-7 and human subcutaneous adipocytes and preadipocytes, respectively. Yuan et al. [43] demonstrated that in mice, corticosterone treatment increased the TNFα expression in MGCs but decreased it in OECs. Furthermore, Zhao et al. [23] found that in mice, while the CRH-induced OEC apoptosis involved both FAS signaling and TNFα signaling, corticosterone-induced OEC apoptosis involved only the FAS, but not the TNFα, signaling. Thus, the effect of glucocorticoids on TNFα expression may vary between species and/or cell types.

The present study demonstrated that treatment with ACTH did not have any detrimental effect on pig embryos, nor OECs, although pig embryos expressed the ACTH receptor. Gong et al. [52] observed that culture with ACTH did not affect oocyte competence and embryo development in the pig, nor that in the mouse. Furthermore, they found that the ACTH receptor was expressed and ACTH-activated protein kinase A in both cumulus-denuded oocytes and CCs. Thus far, we could not find any documentation for the detrimental effects of ACTH on any type of cells.

In this study, the effective concentrations of CRH and cortisol that caused the significant apoptosis of pig OECs were higher than peak serum concentrations reported in stressed pigs. Du et al. [53,54] observed no effect on pig OEC apoptosis by in vitro treatment with cortisol at a stress-induced concentration (2.5 × 10^−7^ M). Our previous study [52] demonstrated that although culture with either 1 × 10^−8^ M CRH or 1 mg/mL cortisol did not affect pig oocyte development into blastocysts, culture with both significantly decreased blastocyst rates. The present results showed that although culture with neither stress-induced concentration of CRH (1 × 10^−8^ M) nor that of cortisol (4 × 10^−7^ M) alone increased apoptosis, culture with both 1 × 10^−8^ M CRH and 4 × 10^−7^ M cortisol significantly increased early and late apoptosis of pig OECs. Thus, the results suggest that in vivo, stresses might increase the release of multiple hormones that work together to induce a symptom at lower concentrations than in vitro in the presence of only a single hormone.

**Figure 8:**
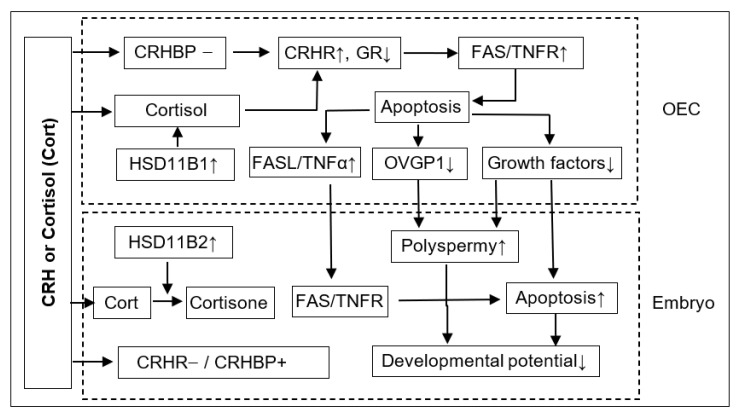
Possible pathways by which corticotrophin-releasing hormone (CRH) or cortisol (Cort) impairs preimplantation development of pig embryos. Please refer to the last paragraph of the Discussion for detailed explanations for the figure. “−”: Not expressed; “+”: Expressed; “↑”: Increased; “↓”: Decreased.

In summary, this study shows that although ACTH showed no effect on pig embryo development, CRH and cortisol impaired preimplantation embryo development and increased polyspermy indirectly by inducing the apoptosis of OECs. Thus, after the treatment of OECs with CRH or cortisol, while the expression level of GR decreased, that of CRHR1, FAS and TNFR1 increased significantly, which would lead to apoptosis (Figure 8). The apoptotic OECs produced more FASL and TNFα but less growth factors and OVGP1. The FASL and TNFα would interact with FAS and TNFR1, respectively, on the embryo, which, together with a desperate shortage of growth factors, would trigger the apoptosis of embryos. The increased polyspermy was associated with a decrease in OVGP1 and growth factors. Both apoptosis and polyspermy would contribute to the decreased developmental potential of embryos. Pig preimplantation embryos did not express CRHR1 but did express CRHBP, which might lead to their insensitivity to CRH. Pig preimplantation embryos expressed more HSD11B2 than HSD11B1, which converts cortisol to inactive cortisone and might contribute to their tolerance to cortisol. Furthermore, when used at a stress-induced physiological concentration, while culture with either CRH or cortisol alone showed no effects, culture with both significantly increased OECs apoptosis, suggesting that in vivo, both hormones may work together to induce apoptosis of pig OECs.

## 5. Conclusions

The results of this study demonstrate that CRH and cortisol impair pig fertilization and preimplantation embryo development indirectly by inducing OEC apoptosis via the activation of the FAS and TNFα systems. However, ACTH did not show any detrimental effect on pig embryos, nor on OECs. The data are important for us to understand the mechanisms by which stress impairs reproduction and to take measures to ameliorate the harmful effects of stress on fertilization and embryo development.

## Figures and Tables

**Table 1:** Oligonucleotide primer sequences used for real-time PCR in this study.

cDNA	Oligonucleotide Sequences (5′-3′)	Amplified Product Size (bp)
*IGF1*	F: CTTGGCCCTGTGCTTGCTCT R: ATCCACGATGCCCGTCTGT	178
*BDNF*	F: GAACTACCCAGTCGTATGTGC R: ATCTTCCCCTCTTAATGGTCA	117
*TGFb1*	F: TGATGTCACCGGAGTTGTGC R: CGGCCAGAATTGAACCCGTTA	135
*CRHBP*	F: ACCATCCATTACGACCGAGT R: AGTAAACCTTCCATTTGGGGTCTG	306
*GAPDH*	F: AAACCTGCAAAATATGATGA R: GTGGTCCAGGGGCTCTTACT	273
*HSD11B1*	F: CGTCACATTACCTCACGGGT R: ACCGAAGTTACAGCCACCAG	107
*HSD11B2*	F: GGCAGTGAAAAACGTGGACC R: TGCCCATTCAAGTGCTCGAT	114
*BCL2*	F: AGGGCATTCAGTGACCTGAC R: CGATCCGACTCACCAATACC	193
*BAX*	F: AGTGGCGGCCGAAATGTTTG R: CAGCAGCCGATCTCGAAGGA	166
*ACTB*	F: CGTGCGGGACATCAAGGA R: AGGAAGGAGGGCTGGAAGA	177

## Data Availability

The data presented in this study are available from the corresponding author upon request.
